# SOS2 modulates the threshold of EGFR signaling to regulate osimertinib efficacy and resistance in lung adenocarcinoma

**DOI:** 10.1002/1878-0261.13564

**Published:** 2024-01-18

**Authors:** Patricia L. Theard, Amanda J. Linke, Nancy E. Sealover, Brianna R. Daley, Johnny Yang, Katherine Cox, Robert L. Kortum

**Affiliations:** ^1^ Department of Pharmacology and Molecular Therapeutics Uniformed Services University of the Health Sciences Bethesda MD USA

**Keywords:** acquired resistance, EGFR‐TKI, osimertinib, PI3K, RAS, SOS2

## Abstract

Son of sevenless 1 and 2 (SOS1 and SOS2) are RAS guanine nucleotide exchange factors (RasGEFs) that mediate physiologic and pathologic receptor tyrosine kinase (RTK)‐dependent RAS activation. Here, we show that SOS2 modulates the threshold of epidermal growth factor receptor (EGFR) signaling to regulate the efficacy of and resistance to the EGFR tyrosine kinase inhibitor (EGFR‐TKI) osimertinib in lung adenocarcinoma (LUAD). *SOS2* deletion (*SOS2* ^*KO*^) sensitized *EGFR*‐mutated cells to perturbations in EGFR signaling caused by reduced serum and/or osimertinib treatment to inhibit phosphatidylinositol 3‐kinase (PI3K)/AKT pathway activation, oncogenic transformation, and survival. Bypassing RTK reactivation of PI3K/AKT signaling represents a common resistance mechanism to EGFR‐TKIs; *SOS2* ^*KO*^ reduced PI3K/AKT reactivation to limit osimertinib resistance. In a forced HGF/MET‐driven bypass model, *SOS2* ^*KO*^ inhibited hepatocyte growth factor (HGF)‐stimulated PI3K signaling to block HGF‐driven osimertinib resistance. Using a long‐term *in situ* resistance assay, most osimertinib‐resistant cultures exhibited a hybrid epithelial/mesenchymal phenotype associated with reactivated RTK/AKT signaling. In contrast, RTK/AKT‐dependent osimertinib resistance was markedly reduced by *SOS2* deletion; the few *SOS2* ^*KO*^ cultures that became osimertinib resistant primarily underwent non‐RTK‐dependent epithelial–mesenchymal transition (EMT). Since bypassing RTK reactivation and/or tertiary *EGFR* mutations represent most osimertinib‐resistant cancers, these data suggest that targeting proximal RTK signaling, here exemplified by *SOS2* deletion, has the potential to delay the development osimertinib resistance and enhance overall clinical responses for patients with *EGFR*‐mutated LUAD.

## Graphical abstract

RTK‐dependent PI3K/AKT activation, associated with a hybrid epithelial/mesenchymal phenotype, is a hallmark of EGFR‐TKI‐resistant lung adenocarcinoma (LUAD). This study shows that *SOS2* deletion reduces the threshold for PI3K/AKT inhibition, effectively reducing the frequency of RTK/AKT‐pathway‐driven EGFR‐TKI‐resistant cultures. These findings suggest that proximal RTK pathway inhibition has the potential to enhance clinical responses for patients with EGFR‐mutated LUAD.

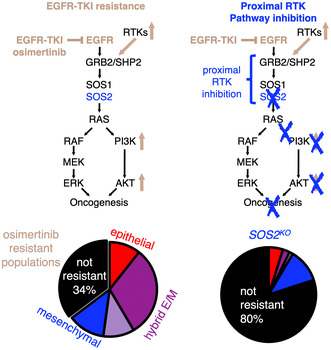

**Abbreviations**
ANOVA: analysis of variance
AUC: area under the curve
CDMRP: Congressionally Directed and Mandated Research Program
EC50: half maximal effective concentration
EGF: epidermal growth factor
EGFR: epidermal growth factor receptor
EGFR‐TKI: epidermal growth factor receptor – tyrosine kinase inhibitor
EMT: epithelial‐mesenchymal transition
ERK: extracellular signal‐regulated kinase
FGFR: fibroblast growth factor receptor
HER2/3: human epidermal growth factor receptors 2/3
HGF: hepatocyte growth factor
HRAS: Harvey‐RAS oncogene
HSP90: heat shock protein 90
Hybrid E/M: hybrid epithelial/mesenchymal
IGF1R: insulin like growth factor 1 receptor
KRAS: Kirsten‐RAS oncogene (protein)
LUAD: lung adenocarcinoma
MEF: mouse embryo fibroblast
MEF: mouse embryonic fibroblast
MEK: MAP/ERK Kinase
NIH: National Institutes of Health
NT: non‐targeting
PI3K: phosphatidylinositol 3‐kinase
RAF: “rapidly accelerated fibrosarcoma,” protein kinase that activates MEK
RAS: Small GTPase, named for Rat Sarcoma viral oncogene
RASGEF: RAS Guanine Nucleotide Exchange Factor
RIPA: radioimmunoprecipitation assay (buffer)
RTK: receptor tyrosine kinase
SCLC: small cell lung cancer
sgRNA: single‐guide RNA (Ribonucleic acid)
SHP2: SH2 domain‐containing protein tyrosine phosphatase‐2
SOS1: son of Sevenless 1
SOS2: son of Sevenless 2
WCL: whole cell lysate

## 1 Introduction

Lung cancer is the leading cause of cancer death; lung adenocarcinoma (LUAD) is the most common subtype of lung cancer [1]. LUAD is primarily a disease of hyperactivated receptor tyrosine kinase (RTK)/RAS signaling, and 75–90% of LUADs harbor oncogenic driver mutations in RTK/RAS pathway members [2, 3, 4]. Activating epidermal growth factor receptor (EGFR) mutations drive oncogenesis in 15–30% of LUADs and are the major cause of LUAD in never‐smokers [1]. For patients with *EGFR‐*mutated LUAD, first (gefitinib and erlotinib), second (afatinib and dacomitinib), and third (osimertinib) generation EGFR‐TKIs (tyrosine kinase inhibitors) have revolutionized cancer treatment. However, despite markedly enhancing survival of patients with *EGFR*‐mutant tumors, resistance to EGFR‐TKIs invariably emerges. For first ‐generation EGFR‐TKIs gefitinib and erlotinib, resistance is primarily driven by either mutation of the drug‐binding site (T790M; 60%) or oncogenic shift to alternative RTKs (15–30%). The third‐generation EGFR‐TKI osimertinib was developed to target T790M‐mutated EGFR; osimertinib showed enhanced progression‐free [5] and overall survival [6] compared to first‐ and second‐generation EGFR‐TKIs and is now the first‐line treatment in *EGFR*‐mutated LUAD. However, despite the increased effectiveness of osimertinib, resistance invariably emerges.

Similar to first‐generation EGFR‐TKI resistance, osimertinib resistance can be driven by both EGFR‐dependent and EGFR‐independent mechanisms; however, unlike first‐generation EGFR‐TKIs EGFR‐independent mechanisms predominate [7, 8, 9, 10]. While the frequency and types of resistance may depend on whether osimertinib was used as first‐line therapy or second‐line therapy after a patient developed resistance to first‐generation EGFR‐TKIs, the most common EGFR‐independent resistance mechanisms involve reactivation of the RTK/RAS/effector pathway [7] via enhanced signaling through parallel RTKs [7, 8, 9, 10, 11, 12, 13, 14, 15, 16]. While individual RTK inhibitors may be beneficial in cancers whose resistance is driven by a specific RTK (MET, AXL, HER2/3, FGFR), broad inhibition of RTK signaling is likely required to enhance osimertinib efficacy and delay therapeutic resistance [7, 8, 9, 10, 11, 12, 13, 14, 15, 16]. Alternatively, a subset of osimertinib‐resistant tumors acquires resistance through histologic transformation via either epithelial‐to‐mesenchymal transformation (EMT) or transition to small cell lung cancer (SCLC). EMT is a dynamic process by which epithelial cells acquire mesenchymal characteristics via changes both in gene transcription and post‐translational regulatory mechanisms and is often characterized by the loss of E‐cadherin and an increase in Vimentin abundance [17]. EMT is a common feature in RTK/RAS pathway independent osimertinib resistance, and targeting EMT via the transcription factors TWIST1 [18] or Snail [19] re‐sensitizes osimertinib‐resistant cells to osimertinib.

The RASGEFs Son of Sevenless 1 and 2 (SOS1 and SOS2) mediate RTK‐stimulated RAS activation and represent common proximal RTK pathway intermediates whose inhibition has the potential to delay therapeutic resistance driven by RTK reactivation [4, 20]. Although SOS1 and SOS2 were previously considered poor candidates for therapeutic intervention due to their low oncogenic potential, recent studies showed that both SOS1 and SOS2 may be important therapeutic targets in *EGFR*‐ and *KRAS‐*mutated cancers [21, 22, 23, 24, 25, 26, 27]. While there are currently no SOS2 inhibitors, SOS1 inhibitors BAY‐293 and BI‐3406 show strong synergy with EGFR‐TKIs [24, 27], KRAS^G12C^ [21, 28], and MEK [22, 29, 30] inhibitors to inhibit survival of *EGFR*‐ or *KRAS*‐mutated LUAD cells [4]. Based on these studies, SOS1 inhibitors are currently in Phase I/II trials for treating *KRAS*‐mutated cancers both as a single agent and in combination with KRAS^G12C^ [NCT04185883; NCT04975256; NCT05578092] or MEK [NCT04111458] inhibitors.

SOS1 and SOS2 have both unique and overlapping roles in regulating physiologic and pathologic RTK/RAS signaling [25, 26, 31, 32, 33, 34, 35, 36, 37, 38, 39, 40, 41]. *Sos1* ^*−/−*^ mice showed embryonic lethality [41] whereas *Sos2* ^*−/−*^ mice were phenotypically normal [42] suggesting differential functionality during embryogenesis. In contrast, *Sos1* KO is well tolerated in adult mice but leads to lethality in a *Sos2* ^*−/−*^ background [33] suggesting some level of functional redundancy. Further, while both T cell [33, 38, 43] and B cell [33] development were decreased in *Sos1* ^*−/−*^ mice, combined *Sos1/2* deletion had a more dramatic effect on development of both lymphocyte populations.

SOS1 and SOS2 show high sequence identity (65%) and similarity (75%) in their N‐terminal domains, but this similarity is markedly reduced (40%) in their C‐terminal proline‐rich regions responsible for GRB2 binding [34, 42, 44] contributing to an increased affinity of SOS2 for GRB2 compared to SOS1 [45]. SOS1, but not SOS2, is subject to both positive and negative feedback regulation. Active RAS^GTP^ binds an allosteric pocket on SOS1 that relieves SOS1 autoinhibition and increases catalytic activity [46, 47, 48], setting up a RAS^GTP^−SOS1−WT RAS positive feedback loop that is not active for SOS2 [26]. SOS1 is also subjected to negative feedback phosphorylation and inactivation by ERK [49] and RSK1 [50], so that SOS1, but not SOS2, activity is curtailed by RAF/MEK/ERK feedback inactivation. SOS1 and SOS2 further have differential effects on activation of RAF/MEK/ERK versus PI3K/AKT effector pathways. SOS1 is a critical regulator of RAF/MEK/ERK signaling in both physiologic [33, 38, 39, 41, 43] and oncogenic [21, 22, 24, 27, 28, 29, 34, 51] settings. In contrast, SOS2 regulates RTK‐PI3K signaling to regulate survival of both epidermal stem cell survival [32] and *KRAS*‐mutated cancer cells [25, 26]. For a comprehensive discussion of the independent and combined roles of SOS1 and SOS2, see [31, 34].

Here we show that SOS2 modulates the threshold of EGFR signaling to regulate the efficacy of and resistance to osimertinib in *EGFR*‐mutated LUAD cells. Using mouse embryonic fibroblasts (MEFs) expressing mutated EGFR proteins, we found that mutant EGFR‐driven transformation was more sensitive to perturbations in the level of EGFR stimulation in *Sos2* ^*−/−*^ cells compared to WT controls. *Sos2* ^*−/−*^ cells showed reduced mutant EGFR‐driven transformation that was inhibited by low levels of EGFR‐TKI treatment and restored by exogenous EGF stimulation. We observed similar results in *EGFR*‐mutated LUAD cells. 3D spheroid growth and survival were more sensitive perturbation of RTK signaling caused by reduced serum conditions and/or treatment with the third‐generation EGFR‐TKI osimertinib in *SOS2* ^*KO*^ cells compared to non‐targeting controls.

RTK pathway reactivation represents a common mechanism driving resistance to EGFR‐TKIs including osimertinib [4, 7, 8, 9, 10, 11, 12, 13, 14, 15, 16], and RTK‐dependent PI3K/AKT activation is a common hallmark of EGFR‐TKI resistance [52, 53]. Using a forced HGF/MEK‐driven bypass model, we found that *SOS2* ^*KO*^ limited HGF‐stimulated AKT signaling and blocked HGF‐driven recalcitrance to osimertinib therapy. Using long‐term *in situ* resistance assays (ISRAs) [54], we found that a majority of osimertinib‐resistant cultures exhibited a hybrid epithelial/mesenchymal phenotype associated with reactivated RTK/AKT signaling. In contrast, *SOS2* deletion markedly reduced the frequency cultures able to obtain osimertinib resistance, with the few resistant *SOS2* ^*KO*^ cultures that did emerge doing so primarily by undergoing non‐RTK dependent EMT. Since bypass RTK reactivation and/or tertiary *EGFR* mutations represent the majority of osimertinib‐resistant cancers, these data suggest that targeting proximal RTK signaling, represented here by *SOS2* deletion, has the potential to prolong the window of therapeutic efficacy for patients with *EGFR*‐mutated LUAD treated with osimertinib.

## 2 Materials and methods

### 2.1 Cell culture

Cell lines were cultured at 37 °C and 5% CO_2_. HCC827, NCI‐H1975, PC9, and PC9‐TM cells were maintained in Roswell Park Memorial Institute medium (RPMI), and immortalized *Sos2* ^−/−^ mouse embryo fibroblasts (MEFs) [26] were maintained in Dulbecco's Modified Eagles Medium (DMEM), each supplemented with 10% fetal bovine serum and 1% penicillin–streptomycin. NCI‐H1975 (RRID:CVCL_1511), HCC827 (RRID:CVCL_2063), and PC9 (RRID:CVCL_B260) cells were obtained from Udayan Guha, NIH. PC9‐TM cells [55] were obtained from Julian Downward, Francis Crick Institute. Cell lines were authenticated by STR profiling in the past 3 years and confirmed as mycoplasma negative. For 2D signaling experiments, cells were seeded in 10 cm dishes at 1.2 × 10^6^ cells per dish. 24 h post‐plating, cells were treated with inhibitor for 6 h and then collected for cell lysis and Western blot analysis. For 3D signaling experiments, cells were seeded in 24‐well micropatterned AggreWell 400 low‐attachment culture plates (STEMCELL Technologies, Vancouver, BC, Canada, #34415) at 1.2 × 10^6^ cells per well in 2 mL of medium. 24 h post‐plating, half of the media was carefully replaced with fresh media to not disturb the spheroids. At 48 h, 1 mL media was removed and replaced with 2× inhibitor. Cells were treated with inhibitor for 6 h and then collected for cell lysis and Western blot analysis.

### 2.2 Cell lysis and Western blot analysis

Cells were lysed in RIPA buffer (1% NP‐40, 0.1% SDS, 0.1% Na‐deoxycholate, 10% glycerol, 0.137 m NaCl, 20 mm Tris pH [8.0], protease (Biotool #B14002) and phosphatase (Bimake.com, Houston, TX, USA, #B15002) inhibitor cocktails) for 20 min at 4 °C and spun at 8600 ***g*** for 10 min. Clarified lysates were boiled in SDS sample buffer containing 100 mm DTT for 10 min prior to Western blotting. Proteins were resolved by sodium dodecyl sulfate‐polyacrylamide (Novex precast, ThermoFisher, Waltham, MA, USA) gel electrophoresis and transferred to nitrocellulose membranes. Western blots were developed by multiplex Western blotting using anti‐SOS2 (Santa Cruz, Houston, TX, USA, sc‐258; 1 : 500), anti‐β‐actin (Sigma, St. Louis, MO, USA, AC‐15; 1 : 5000), anti‐pEGFR (Cell Signaling 3777; 1 : 1000), anti‐EGFR (Cell Signaling, Danvers, MA, USA, 4267; 1 : 1000), anti‐pERK1/2 (Cell Signaling, 4370; 1 : 1000), anti‐ERK1/2 (Cell Signaling 4696; 1 : 1000), anti‐pAKT Ser^473^ (Cell Signaling 4060; 1 : 1000), anti‐AKT (Cell Signaling 2920; 1 : 1000), anti‐HSP90 (Santa Cruz, sc‐7947, 1 : 1000), anti‐α‐tubulin (Abcam, Boston, MA, USA, ab89984; 1 : 2000), Vimentin (Cell Signaling 5741; 1 : 1000), and E‐cadherin (Cell Signaling 14 472; 1 : 1000) primary antibodies. Anti‐mouse and anti‐rabbit secondary antibodies conjugated to IRDye680 or IRDye800 (LI‐COR; Lincoln, NE, USA, 1 : 10 000) were used to probe primary antibodies. Western blot protein bands were detected and quantified using the Odyssey system (LI‐COR). For quantification of SOS2 abundance, samples were normalized to either β‐actin or HSP90. For quantification of pAKT, pERK, and pEGFR, samples were normalized to a weighted average of total AKT, total ERK1/2, total EGFR, HSP90, and β‐actin as we had previously done [26] and as first reported in [56]. For quantification of pAKT, pERK, E‐cadherin, and Vimentin, samples were normalized to a weighted average of total ERK1/2, total AKT, HSP90, and β‐actin [56]. Classification of samples as pAKT^hi^ versus pAKT^low^ was determined relative to the abundance of pAKT/total protein in parental H1975 cells. Cultures with pAKT/total protein abundance greater than what was observed in parental H1975 cells were classified as pAKT^hi^, whereas cultures with pAKT/total protein abundance less than what was observed in parental H1975 cells were classified as pAKT^low^.

### 2.3 Production of recombinant lentiviruses

Lentiviruses for both sgRNA studies (NT versus *SOS2* ^*KO*^) [26] and mutant EGFR expression [57, 58] were produced by co‐transfecting MISSION lentiviral packaging mix (Sigma) into 293 T cells using Mirus *Trans*IT^®^‐Lenti transfection reagent (Mirus Bio, Madison, WI, USA, # MIR6605) in Opti‐MEM (Thermo Scientific, Waltham, MA, USA, #31‐985‐062). At 48 h post‐transfection, viral supernatants were collected and filtered. Viral supernatants were then either stored at −80 °C or used immediately to infect cells in combination with polybrene at 8 μg·mL^−1^. 48 h post‐infection, cells were selected in 4 μg·mL^−1^ Puromycin (Invitrogen, Waltham, MA, USA).

### 2.4 Transformation studies

MEFs expressing mutant EGFR were seeded in 0.32% Nobel agar at 2 × 10^4^ cells per 35‐mm dish to assess anchorage‐independent growth. Soft agar colonies were counted 28 days after seeding. For all other cell lines spheroid growth was assessed in ultra‐low attachment 96‐well round‐bottomed plates (Corning, Glendale, AZ, USA, Costar #7007, S‐BIO PrimeSurface #MS‐9096UZ, or Nunc Nucleon Sphera microplates ThermoFisher # 174929), cells were seeded at 500 cells per well. Cell number was assessed in parallel plates at 0, 7, 14, and 21 days using CellTiter‐Glo^®^ 2.0 reagent.

### 2.5 sgRNA studies

Cells were infected with lentiviruses (pLentiCRISPRv2 [59]) expressing Cas9 and either a non‐targeting (NT) single guide RNA (sgRNA) or a SOS2‐targeted sgRNA (SOS2‐9) as previously described [26, 27]. Cell lysates were probed for SOS2, and only cell populations (not clones) showing greater that 80% loss of SOS2 protein abundance within the overall population were used. Independent infections were used for replicate experiments.

### 2.6 Inhibitor studies

For 2D adherent studies cells were seeded at 500–1000 cells per well in 100 μL in the inner‐60 wells of 96‐well white‐walled culture plates (PerkinElmer, Waltham, MA, USA) and allowed to attach for 48 h prior to drug treatment. Cells were treated with drug for 96 h (HGF‐stimulation studies) or 120 h (MEFs) prior to assessment of cell viability using CellTiter‐Glo^®^ 2.0. For 3D spheroid studies cells were seeded at 500–1000 cells per well in 100 μL in the inner‐60 wells of 96‐well ultra‐low attachment round‐bottomed plates (Corning #7007) or Nunc Nucleon Sphera microplates (ThermoFisher # 174929) and allowed to coalesce as spheroids for 48–72 h prior to drug treatment. For HGF‐stimulation studies, cells were treated with osimertinib ± HGF (30 ng·mL^−1^) for 96 h prior to the assessment of cell viability using CellTiter‐Glo^®^ 2.0. For transformation studies at different serum concentrations, cells were treated with increasing doses of osimertinib for 7 (PC9) or 21 (H1975, HCC827) days. In all studies parallel plates were assessed for cell viability at the time of drug treatment (day 0) to calculate the fold‐change in cell number.

### 2.7 *In situ* resistance assays

*In situ* resistance assays were performed as previously described [54]. Briefly, NT and *SOS2* ^*KO*^ cells were seeded at 250 cells per well in the inner 60 wells of replicate 96‐well tissue culture plates and allowed to adhere for 24 h prior to treatment with 50, 150, or 300 nm osimertinib, each plate representing a single drug treatment trial. Plates were fed and wells were scored weekly, with wells reaching > 50% confluence scored as resistant. A subset of resistant NT and *SOS2* ^*KO*^ H1975 wells were continuously cultured in osimertinib and expanded prior to whole‐cell lysis and assessment by Western blotting.

### 2.8 Statistical analysis

For transformation studies and assessment of differences between AOC and EC_50_ of dose–response curves, statistical significance was determined by two‐way ANOVA followed by a Bonferroni correction to adjust for multiple comparisons using prism 9 (Graphpad Software, Boston, MA, USA). Non‐linear fitting to determine EC_50_ and AOC from dose–response experiments was performed using prism 9. For resistance assays, data were plotted as Kaplan–Meier survival curves, and significance was assessed by pairwise comparisons of Kaplan–Meyer Meier curves using prism 9. Comparison of the frequencies of pAKT^hi^ versus pAKT^low^ populations in osimertinib‐resistant NT and *SOS2* ^*KO*^ cells was performed via contingency analysis in prism 9, and statistical significance was determined via chi‐square test.

## 3 Results

### 3.1 SOS2 mediate mutant EGFR‐dependent transformation

**Fig. 1:**
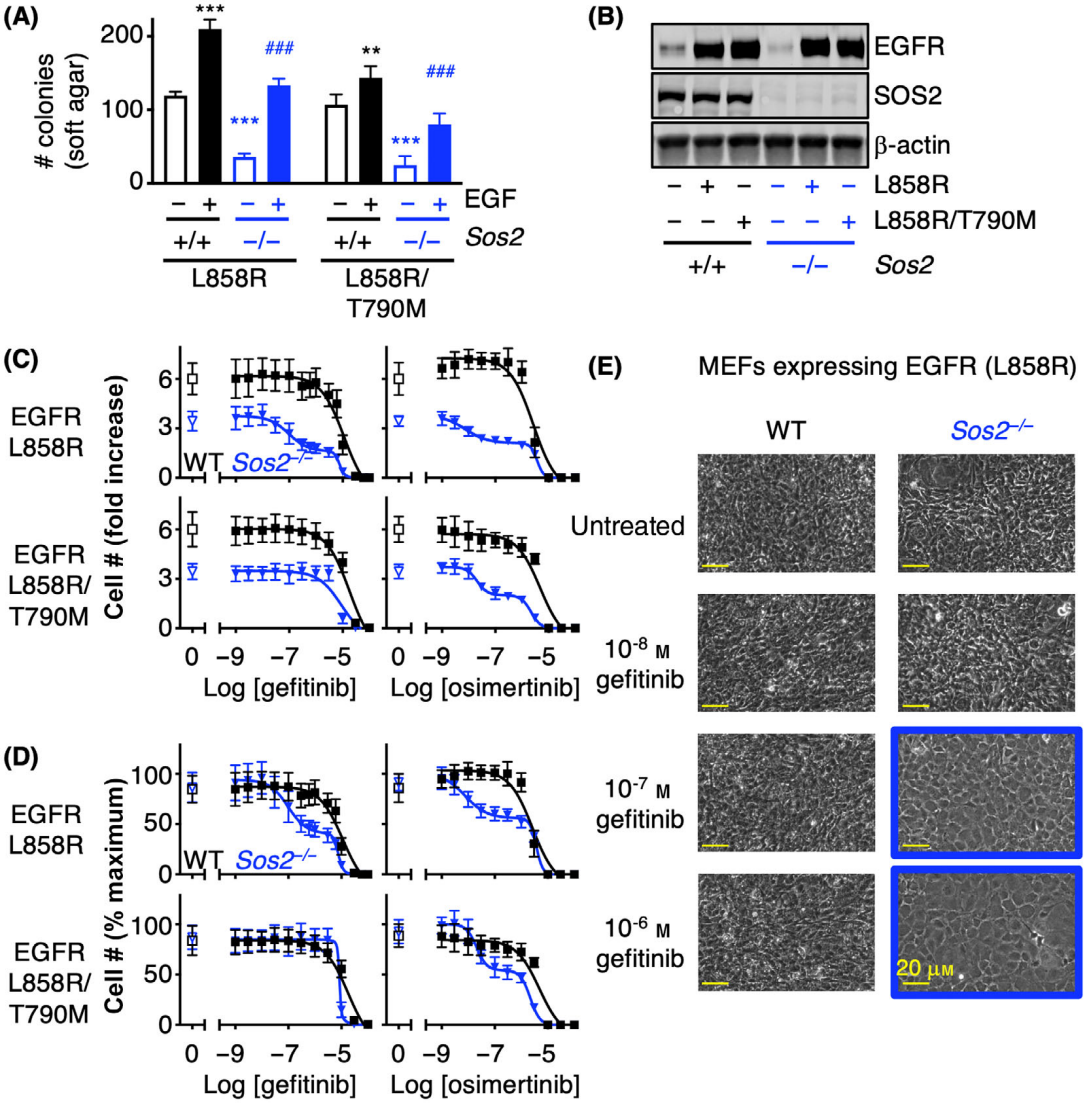
*Sos2* deletion synergizes with epidermal growth factor receptor (EGFR) – tyrosine kinase inhibitor (EGFR‐TKI) treatment to inhibit mutant EGFR‐driven transformation. (A) Soft agar assays from WT and *Sos2* ^*−/−*^ mouse embryonic fibroblasts (MEFs) ectopically expressing L858R or L858R/T790M mutated EGFR showing that *Sos2* deletion raises the threshold of EGFR stimulation required to promote anchorage‐independent growth (soft agar) in MEFs expressing first‐generation EGFR‐TKI sensitive (L858R) or resistant (L858R/T790M) mutated EGFR. Data were analyzed by ANOVA with a Bonferroni correction for multiple comparisons and are presented as mean ± SD from *n* = three independent experiments. **P < 0.01, ***P < 0.001 versus NT control (unstimulated); ^###^ *P* < 0.001 versus EGF‐stimulated NT control. (B) Whole‐cell lysates (WCLs) of cells from (A) were analyzed by Western blotting with antibodies specific for EGFR, son of sevenless (SOS2), or β‐Actin. Western blots are representative from *n* = three independent experiments. (C, D) Dose–response curves of WT (black squares) or *Sos2* ^−/−^ (blue inverted triangles) MEFs expressing first‐generation EGFR‐TKI sensitive (L858R) or resistant (L858R/T790M) mutated EGFR from treated with increasing doses of the first‐generation EGFR‐TKI gefitinib (left) or the third‐generation EGFR‐TKI osimertinib (right) for 5 days. *Sos2* ^*−/−*^ MEFs showed a biphasic dose response to EGFR‐TKI treatment in responsive cells. Dose–response curves are normalized to cell number assessed 2 h after plating by CellTitre Glo (C) or to cell number in untreated NT or *Sos2* ^*−/−*^ MEFs at the end of the five‐day experiment (D). Data were analyzed by non‐linear regression and are presented as mean ± SD from *n* = three independent experiments. (E) 10× photographs of post‐confluent *Sos2* ^*+/+*^ or *Sos2* ^*−/−*^ MEFs expressing EGFR (L858R) treated with the indicated dose of gefitinib from (C) showing that transformation (loss of contact inhibition) is blocked at low doses of gefitinib treatment in *Sos2* ^*−/−*^ MEFs. Blue outline indicates conditions where gefitinib treatment restored contact inhibition. All images are scaled equivalently; scale bar represents 20 μm. Photographs are representative from *n* = three independent experiments.

To investigate the role of SOS2 in mutant EGFR‐driven oncogenesis, we assessed anchorage‐independent growth in immortalized WT versus *Sos2* ^−/−^ MEFs [26] expressing either a first‐generation EGFR‐TKI sensitive (L858R) or resistant (L858R/T790M) mutant EGFR in both the absence and presence of EGF stimulation (Fig. 1A,B). EGF stimulation was performed as a large proportion of lung adenocarcinomas show high expression of EGFR ligands [60] and seminal experiments showed that EGF stimulation promoted transformation in cells overexpressing WT EGFR and enhanced transformation in cell expressing oncogenic EGFR mutants [57, 61]. In the absence of exogenous EGF stimulation, we found that SOS2 was a critical modifier of mutant EGFR‐driven transformation (Fig. 1A, open bars). *Sos2* deletion significantly reduced mutant EGFR‐driven transformation in the absence of exogenous EGF by > 75%, revealing a previously uncharacterized role for SOS2 in mutant EGFR‐driven transformation.

Upon EGF stimulation, WT MEFs expressing mutated EGFR proteins showed a 1.5‐ to 2‐fold increase in transformed colonies, confirming a role in ligand‐dependent enhancement of EGFR‐driven oncogenesis. Intriguingly, EGF stimulation partially restored mutant EGFR‐driven transformation in *Sos2* ^−/−^ cells (Fig. 1A). These data suggest that SOS2 may modulate the threshold of EGFR signaling required to promote oncogenesis so that under conditions that EGFR signaling is limiting, SOS2 ablation could limit mutant EGFR‐driven transformation.

### 3.2 SOS2 regulates the threshold of EGFR‐TKI dependent inhibition of oncogenesis

To test the extent to which SOS2 modifies transformation under conditions where EGFR signaling is inhibited, we treated WT versus *Sos2* ^*−/−*^ MEFs expressing both first‐generation EGFR‐TKI sensitive (L858R) and resistant (L858R/T790M) EGFR mutants with increasing doses of either a first (gefitinib) or third (osimertinib) generation EGFR‐TKI and assessed dose‐dependent changes in cell number. MEFs were seeded in 96‐well cell culture plates and grown for 48 h; cells were approximately 50% confluent prior to treatment with EGFR‐TKI. This cell density allows for the assessment of post‐confluent cell growth due to loss of contact inhibition; untreated WT MEFs expressing mutant EGFR showed a roughly 6‐fold increase in cell number over the five‐day period, whereas *Sos2* ^*−/−*^ cells showed only a 3‐fold increase in cell number due to reduced transforming growth (Fig. 1C). Dose–response curves were plotted both as fold‐change in cell number compared to day 1 (Fig. 1C) to assess inhibition of proliferation/transformation and as % of maximum growth for each cell line (Fig. 1D) to allow a better visual assessment of EC_50_ values between the cell lines. In WT MEFs expressing EGFR (L858R), gefitinib and erlotinib inhibited cell outgrowth at very high levels of drug (EC_50_ ~ 10 μm), indicative of general toxicity rather than on‐target inhibition (Fig. 1C,D). In contrast, *Sos2* ^−/−^ cells expressing a first‐generation EGFR‐TKI sensitive mutant [EGFR (L858R)] showed a biphasic response to both gefitinib and osimertinib with the first inflection approximately 2‐log lower than the toxic dose for either drug (Fig. 1C,D). *Sos2* ^−/−^ cells expressing a first‐generation EGFR‐TKI resistant mutant [EGFR (L858R/T790M)] were unresponsive to gefitinib but showed a similar biphasic response to osimertinib treatment (Fig. 1C,D). To confirm that the first EGFR‐TKI‐dependent inhibition of cell number in *Sos2* ^−/−^ cells was due to inhibiting transformation, WT and *Sos2* ^−/−^ MEFs expressing EGFR (L858R) were treated with increasing doses of gefitinib for 2 weeks (1‐week post‐confluence) and transformation was assessed by loss‐of‐contact inhibition. WT MEFs showed loss‐of‐contact inhibition (transformation) at gefitinib doses up to 1 μm (Fig. 1E). In contrast, *Sos2* ^−/−^ MEFs treated with ≥ 100 nm gefitinib were contact inhibited and grew as a monolayer (Fig. 1E). These data suggest SOS2 may be an important modifier of oncogenic growth and EGFR‐TKI responsiveness in *EGFR*‐mutated cancer cells.

**Fig. 2:**
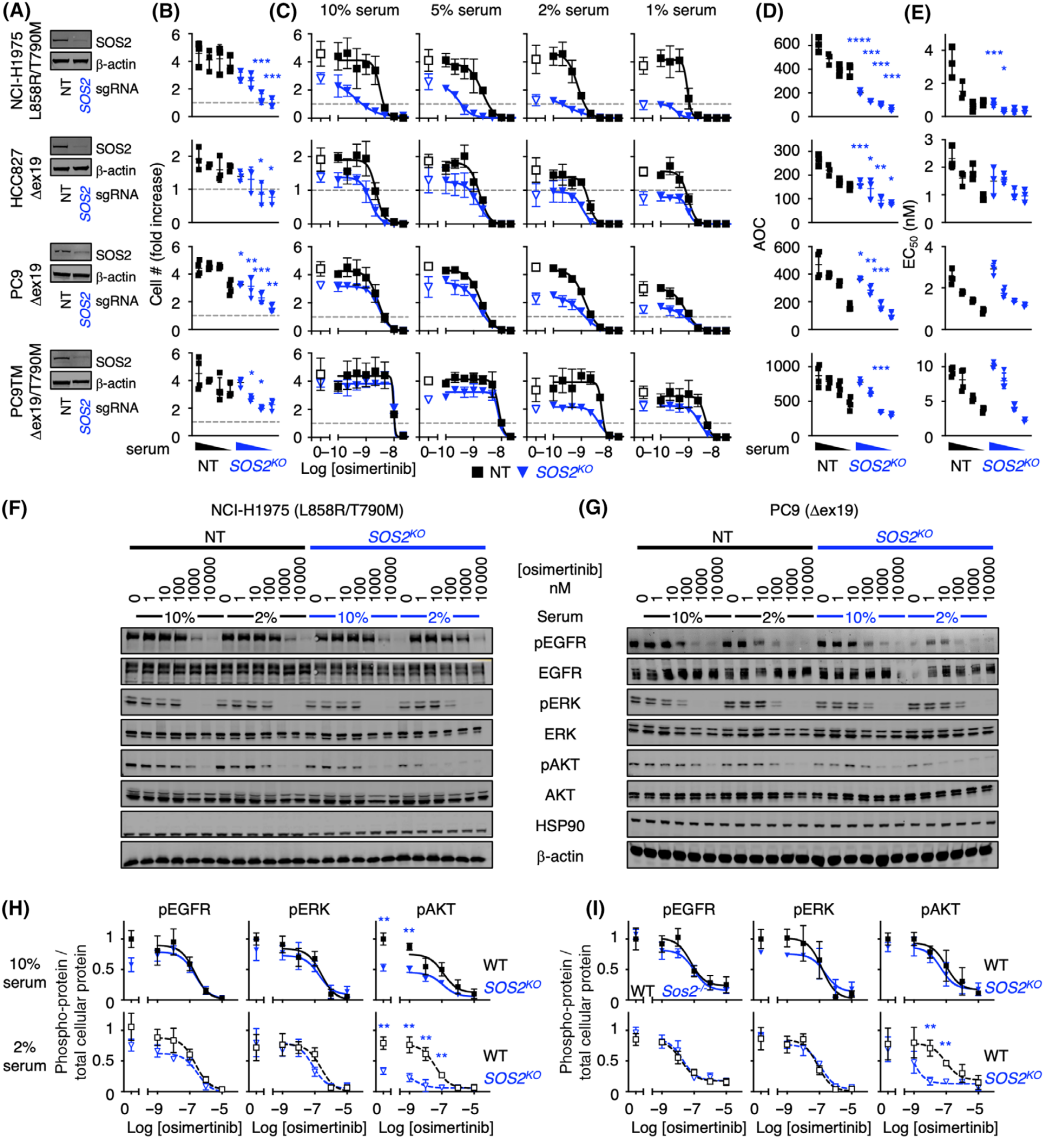
*SOS2* deletion increases the threshold of EGFR stimulated required for oncogenic transformation. (A) Western blots of whole cell lysates (WCLs) showing *SOS2* deletion in pooled populations of H1975, HCC827, PC9, or PC9‐TM cells compared to non‐targeting (NT) controls. (B‐C) 3D spheroid growth under decreasing serum concentrations (10%, 5%, 2%, and 1%) in the absence of epidermal growth factor receptor (EGFR) – tyrosine kinase inhibitor (EGFR‐TKI) treatment (B) or at increasing doses of osimertinib (C) in pooled populations of H1975, HCC827, PC9, or PC9‐TM cells (see labels in A) where *SOS2* has been deleted using CRISPR/Cas9 versus NT controls after 7 (PC9; PC9‐TM) or 21 (H1975; HCC827) days to allow for transforming growth. Data were analyzed by ANOVA with a Bonferroni correction for multiple comparisons (B) or by non‐linear regression (C) and are presented as mean ± SD from *n* = three (H1975, HCC827) or *n* = four (PC9; PC9‐TM) independent experiments. The horizontal dashed line indicates the number of cells plated at the beginning of each experiment. (D, E) area under the curve (AUC) (D) and EC_50_ values (E) for osimertinib dose–response experiments from (C). (F–I) Western blots (F, G) and quantitation of pEGFR, pERK, and pAKT normalized to a weighted average of total protein (H, I) from WCLs of 3D spheroid cultured *SOS2* ^*KO*^ NCI‐H1975 (F, H) or PC9 (G, I) cells versus NT controls treated with increasing doses of osimertinib under high serum (10%) or low serum (2%) conditions for 6 h. Western blots are for pEGFR, EGFR, pERK, ERK, pAKT, AKT, HSP90, and β‐actin. Data were analyzed by ANOVA with a Bonferroni correction for multiple comparisons (B, D, E) or by non‐linear regression (C, H, I) and are presented as mean ± SD from *n* = three (H1975, HCC827) or *n* = four (PC9; PC9‐TM) independent experiments. Western blots (A, F, G) are representative from *n* = three (H1975, HCC827) or *n* = four (PC9; PC9‐TM) independent experiments. *P < 0.05; **P < 0.01; ***P < 0.001 versus NT controls.

We next assessed the extent to which SOS2 regulated the threshold of EGFR signaling to promote oncogenesis in human *EGFR*‐mutated LUAD cells. *SOS2* was deleted in a panel of *EGFR*‐mutated LUAD cell lines (Fig. 2A). 3D spheroid growth was assessed over 21 (H1975, HCC827) or seven (PC9, PC9TM) days at decreasing serum concentrations in either untreated cells (Fig. 2B) or at increasing osimertinib concentrations (Fig. 2C). For all CRISPR experiments, we assessed the effect of *SOS2* deletion from cell populations that showed > 80% decreases in SOS2 protein abundance compared to NT controls; populations were used rather than cell clones to avoid clonal effects not related to *SOS2* ^*KO*^. In non‐targeting (NT) controls, 3D spheroid growth was relatively unhindered when cultured at low serum concentrations (Fig. 2B). In contrast, the effect of *SOS2* deletion on transformation was serum‐dependent. While *SOS2* ^*KO*^ had a modest effect on transformation in 10% serum, the dependence of transformation on SOS2 was more pronounced as serum concentrations decreased so that at 2% or 1% serum *SOS2* ^*KO*^ cells showed a marked inhibition of 3D spheroid growth compared to NT controls (Fig. 2B). These data suggest a critical role for SOS2 in mutant EGFR‐driven transformation under nutrient limiting conditions.

We further assessed the extent to which *SOS2* deletion enhanced osimertinib dose‐dependent inhibition of transformation (above gray line, Fig. 2C) and survival (below gray line, Fig. 2C) in long‐term 3D spheroid cultured LUAD cells. In both NT and *SOS2* ^*KO*^ cells, osimertinib caused a dose‐dependent decrease in transforming growth at osimertinib low doses and inhibited survival at higher doses. However, the effect of *SOS2* deletion on osimertinib‐dependent 3D transformation and survival was more dependent on serum concentration compared to NT controls (Fig. 2C). In 10% serum, *SOS2* ^*KO*^ had a modest effect on osimertinib‐dependent inhibition of transformation compared to NT controls in three of four cell lines (HCC827, PC9, PC9TM), but at lower serum levels *SOS2* ^*KO*^ enhanced osimertinib‐dependent inhibition of transformation and survival as assessed by both an overall decrease in AUC (Fig. 2D) in all four cell lines and an EC_50_ shift in H1975 cells (Fig. 2E) consistent with the marked osimertinib‐dependent effect observed in H1975 cells at all serum concentrations (Fig. 2C).

We further assessed the extent to which *SOS2* ^*KO*^ affected the activation of downstream signaling pathways associated with 3D proliferation and survival in whole‐cell lysates of 3D cultured spheroids. In H1975 and PC9 cells, *SOS2* ^*KO*^ did not alter ERK phosphorylation as a surrogate of RAF/MEK/ERK signaling in either 10% or 2% serum (Fig. 2F–I). In contrast, *SOS2* ^*KO*^ decreased AKT phosphorylation as a surrogate of PI3K/AKT signaling in both untreated and osimertinib‐treated H1975 cells (Fig. 2F,H) and in osimertinib‐treated PC9 cells (Fig. 2G,I) cultured under low serum conditions. These data support previous studies describing the differential preference of SOS2 for promoting EGF‐stimulated PI3K/AKT activation in *KRAS*‐mutated cells [25, 26].

### 3.3 *SOS2* deletion limits the development of osimertinib resistance

**Fig. 3:**
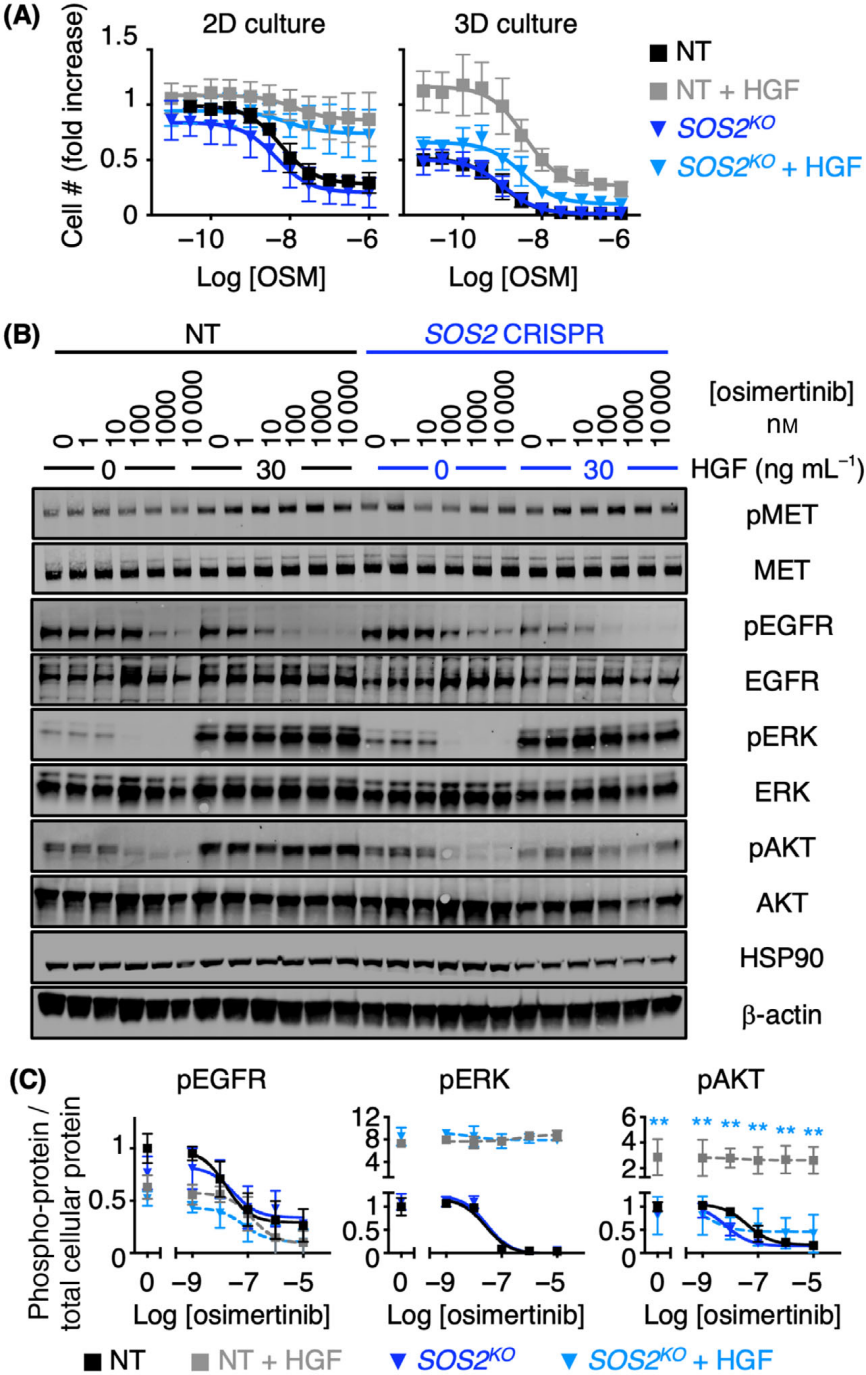
*SOS2* deletion limits hepatocyte growth factor (HGF)‐dependent osimertinib resistance in 3D cultured H1975 cells. (A) Dose–response curves of *SOS2* ^*KO*^ and non‐targeting control (NT) H1975 cells treated with increasing doses of osimertinib ± HGF under 2D adherent (top) or 3D spheroid (bottom) culture conditions for 4 days. Dose–response curves are normalized to cell number assessed 2 h after plating by CellTitre Glo. Data were analyzed by non‐linear regression and are presented as mean ± SD from *n* = three independent experiments. (B) Western blots of whole cell lysates (WCLs) from 3D cultured or SOS2^KO^ H1975 cells versus NT controls treated with increasing doses of osimertinib ± HGF (to bypass EGFR signaling) for 6 h versus NT controls. Western blots are for pMET, MET pEGFR, EGFR, pERK, ERK, pAKT, AKT, HSP90, and β‐Actin. Western blots are representative of *n* = three independent experiments. (C) Quantitation of pEGFR, pERK, and pAKT normalized to a weighted average of total protein from Western blots in B. Data in A and C were analyzed by nonlinear regression and are presented as mean ± SD from *n* = three independent experiments; significance was assessed by ANOVA with a Bonferroni correction for multiple comparisons. **P < 0.01 versus NT controls.

Resistance to EGFR‐TKIs including osimertinib is most often driven by RTK/RAS/PI3K pathway reactivation [7] via either tertiary EGFR mutations or enhanced signaling through parallel RTKs including MET, AXL, HER2/3, and FGFR [8, 9, 10, 11, 12, 13, 14, 15, 16]. Since *SOS2* ^*KO*^ enhanced osimertinib‐dependent inhibition of PI3K/AKT signaling, we hypothesized that SOS2 could be an important regulator of RTK/PI3K‐dependent osimertinib resistance. MET amplification is one of the most common alternative RTK‐dependent EGFR‐TKI resistance mechanisms; MET‐dependent osimertinib resistance can be modeled by exogenous HGF stimulation [62]. To assess the extent to which SOS2 regulates osimertinib resistance driven by alternate RTKs, we assessed osimertinib dose‐dependent inhibition of survival after 4 days of drug treatment in both 2D (adherent) and 3D spheroid cultured NT and *SOS2* ^*KO*^ H1975 cells either in the absence or presence of HGF stimulation (Fig. 3) in 10% serum culture conditions; *SOS2* ^*KO*^ was previously shown to not alter 3D survival after short‐term osimertinib treatment [27]. In H1975 cells cultured in 2D conditions, HGF inhibited osimertinib‐induced decreases in cell number; however, *SOS2* deletion did not significantly alter the sensitivity of cells to osimertinib in either the absence or presence of HGF compared to NT controls (Fig. 3A). In contrast, 3D spheroid‐cultured *SOS2* ^*KO*^ cells showed enhanced osimertinib‐dependent inhibition of survival (Fig. 3A). We further assessed RTK pathway signaling in 3D‐cultured NT and *SOS2* ^*KO*^ cells treated with increasing doses of osimertinib ± HGF (Fig. 3B,C). HGF stimulated MET phosphorylation whereas osimertinib‐dependent inhibition of EGFR phosphorylation was exacerbated in HGF‐stimulated cells, consistent with previous studies showing enhanced pEGFR inhibition in osimertinib‐resistant cells driven by AXL, MET, or IGF1R [16, 63, 64, 65]. pEGFR inhibition was further exacerbated in *SOS2* ^*KO*^ cells, possibly due to either modulation of RTK‐driven compensatory pathways [66] or loss of SOS and GRB2‐dependent oligomerization of EGFR similar to what has been observed for the T cell adaptor LAT [67, 68]. Notably, we observed a marked reduction in pAKT, but not pERK, in HGF‐stimulated *SOS2* ^*KO*^ cells compared to NT controls (Fig. 3B,C). Since *SOS2* ^*KO*^ exclusively inhibited AKT, but not ERK activation, these data suggest that SOS2 is a critical determinant of RTK/PI3K‐dependent osimertinib resistance.

To directly assess the extent to which SOS2 regulates the development of acquired resistance to osimertinib, we used an *in situ* resistance assay [54] that acts as a cell culture model of a multiple‐subject trial to assess resistance to RTK/RAS pathway inhibitors. This hybrid approach combines elements of time‐to‐progression assays [69, 70] and cell outgrowth assays [52, 71, 72] allowing us to monitor the development of *de novo* osimertinib resistance. In this assay, cells are seeded at low density (250 cells per well, < 10% confluent) in the inner 60 wells of multiple 96‐well plates; the inner 60 wells are used to avoid “edge effects” associated with long‐term culture of cells in multi‐well plates. Each plate is then treated with a different dose of drug (or drug combination), so that each plate acts as a 60‐subject arm of a “trial” comparing different treatments. Wells are fed (fresh media/drug) and scored weekly; wells that reached ≥ 50% confluence were scored as resistant to that drug or drug combination. We found that treatment of RTK/RAS pathway mutated cells using a ≥ EC_80_ dose of an oncogene‐targeted therapy [osimertinib (*EGFR*‐mutated), adagrasib (*KRAS* ^*G12C*^‐mutated), sotorasib (*KRAS* ^*G12C*^‐mutated), trametinib (*KRAS*‐mutated), tipifarnib (*HRAS*‐mutated)] modeled acquired resistance *in situ* [54].

**Fig. 4:**
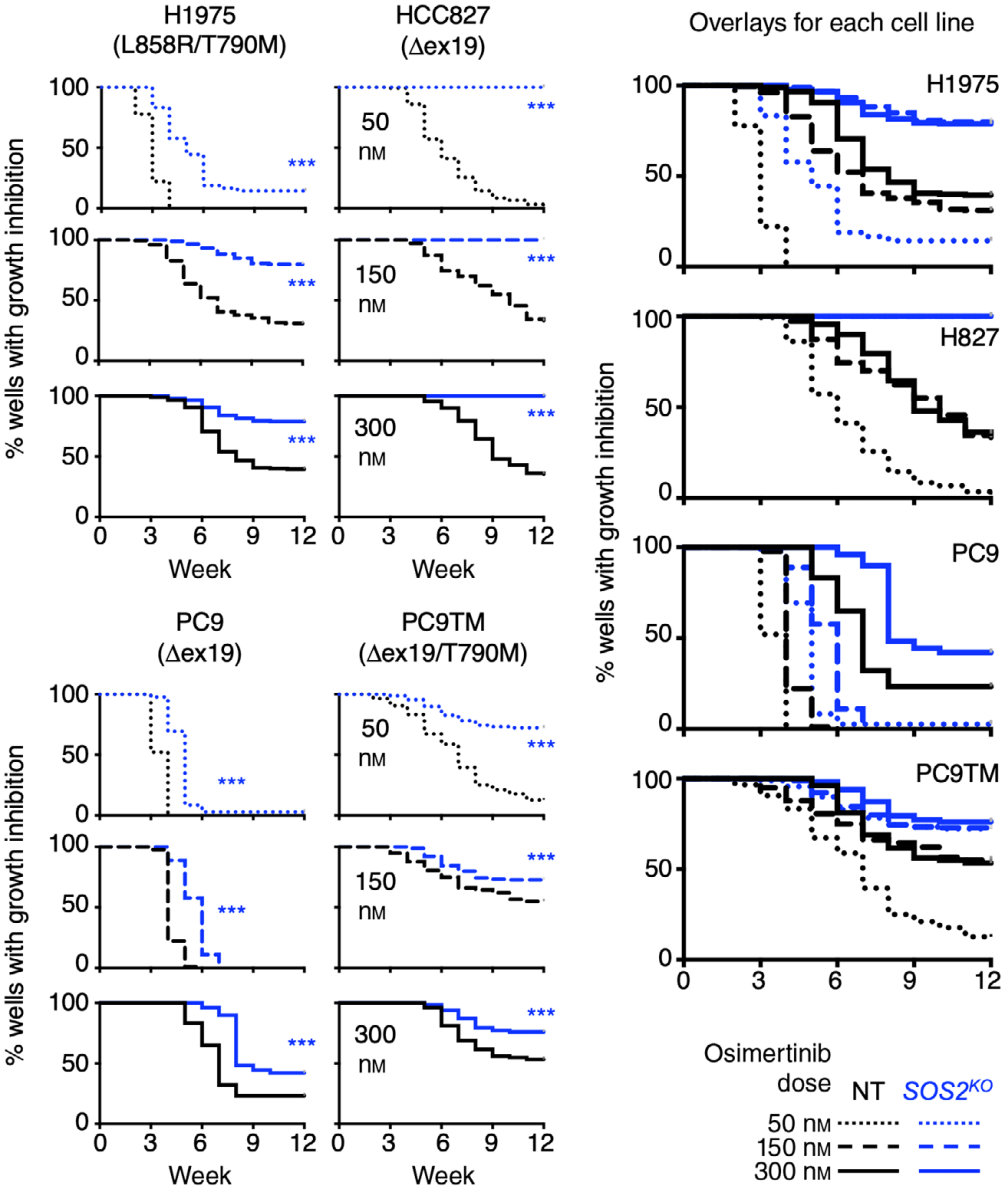
*SOS2* deletion limits osimertinib resistance in cell culture models. Multi‐well resistance experiments in non‐targeting control (NT) (black) versus *SOS2* ^*KO*^ (blue) H1975, HCC827, PC9, and PC9‐TM cells treated with 50 nm (dotted), 150 nm (dashed), or 300 nm (solid) osimertinib. Individual curves for individual osimertinib doses (left) and overlays of all osimertinib doses (right) are shown for each cell line. ***P < 0.001 for *SOS2* ^*KO*^ compared to NT controls. Data were analyzed by Kaplan‐Meyer survival statistics using prism 9 and are pooled from *n* = three independent experiments.

NT and *SOS2* ^*KO*^ H1975, HCC827, PC9, and PC9‐TM cells were seeded at low density in the inner 60 wells of multiple 96 well plates and each plate was treated with a single dose (50–300 nm) of osimertinib. Wells were fed and scored weekly; wells that reached ≥ 50% confluence were scored as osimertinib resistant and data were plotted as a Kaplan–Meier curve (Fig. 4). In cells treated with a ≤ EC_80_ osimertinib dose (50 nm in all cell lines, 150 nm in PC9 cells) that causes reduced proliferation but is insufficient to overcome intrinsic resistance and thus model acquired in NT controls, *SOS2* ^*KO*^ significantly delayed the outgrowth of drug‐treated populations in all four cell lines (Fig. 4, dotted lines). Further, in two cell lines (HCC827 and PC9TM), > 50% of *SOS2* ^*KO*^ cultures remained sensitive to 50 nm osimertinib over 12 weeks of treatment indicating that SOS2 deletion may lower the dose of osimertinib necessary to overcome intrinsic resistance and show therapeutic efficacy. In cells treated with doses of osimertinib sufficient to cause prolonged growth arrest and model drug resistance (150 or 300 nm), *SOS2* ^*KO*^ both delayed the outgrowth of osimertinib‐resistant cells and reduced the overall frequency of wells able to develop osimertinib resistance (Fig. 4, dashed and solid lines). These data suggest that proximal RTK pathway inhibition, achieved here via *SOS2* ^*KO*^, may be a strategy to limit osimertinib resistance.

### 3.4 Osimertinib‐resistant cultures show a hybrid epithelial‐mesenchymal phenotype associated with RTK/PI3K pathway reactivation

RTK pathway reactivation [4, 7, 8, 9, 10, 11, 12, 13, 14, 15, 16], often by simultaneous activation of multiple RTKs [54, 73], represents a common mechanism driving resistance to EGFR‐TKIs including osimertinib. RTK‐dependent PI3K/AKT activation is a common hallmark of EGFR‐TKI resistance [52, 53], and *SOS2* ^*KO*^ reduced HGF‐stimulated PI3K/AKT signaling to inhibit HGF‐mediated osimertinib resistance in *EGFR*‐mutated cells (Fig. 3). Thus, we hypothesized that the reduced frequency with which *SOS2* ^*KO*^ cultures developed osimertinib resistance in long‐term cultures was due to by reduced SOS2‐dependent PI3K signaling, and further that *SOS2* ^*KO*^ cultures would become osimertinib resistant via non‐RTK dependent mechanisms. To determine whether osimertinib‐resistant *SOS2* ^*KO*^ cultures were fundamentally different than NT controls, we expanded 67 NT and 37 *SOS2* ^*KO*^ osimertinib‐resistant populations from H1975 cells treated with 150 or 300 nm osimertinib for ≥ 6 weeks and assessed for RTK pathway reactivation (pERK and pAKT) and markers of EMT (E‐cadherin and Vimentin) by Western blotting of whole cell lysates from adherent (2D) cultures of each osimertinib‐resistant population (Fig. S1). Cultures were expanded in the presence of osimertinib to ensure osimertinib‐resistance, but osimertinib was removed 48 h prior to cell lysis to allow comparison of naïve signaling pathways compared to parental controls. Since we performed our resistance studies in pooled cultures that showed > 90% (but not complete) loss of SOS2 protein, there was the possibility that some osimertinib‐resistant *SOS2* ^*KO*^ cultures may outgrow from a sub‐population of cells that had not deleted *SOS2*. Indeed, four isolated *SOS2* ^*KO*^ colonies showed ≥ 40% SOS2 protein abundance observed in NT controls and were thus excluded from our analysis (crossed out cell lines in Fig. S1). It was also possible that the closely related family member SOS1 could be upregulated to compensate for the loss of SOS2 in our cultures. However, we did not observe a significant increase in SOS1 protein in any osimertinib‐resistant *SOS2* ^*KO*^ resistant cultures compared to parental H1975 controls. These data indicate that other mechanisms account for osimertinib resistance in the 20% of *SOS2* ^*KO*^ cultures showing osimertinib resistance.

**Fig. 5:**
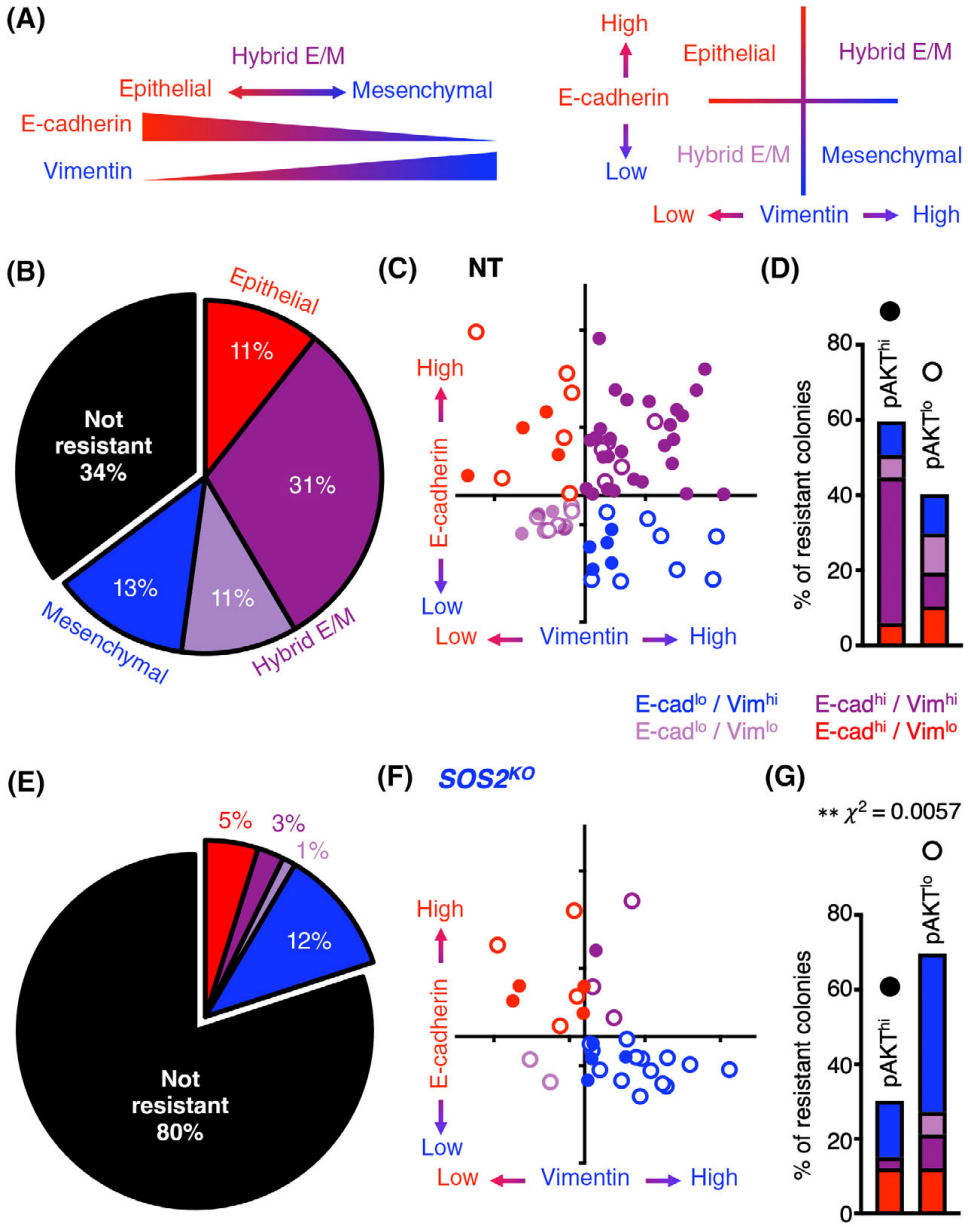
The hybrid epithelial/mesenchymal (E/M) phenotype in osimertinib‐resistant cells is SOS2‐dependent. (A) The epithelial‐to‐mesenchymal transition (EMT) can be characterized by loss of the epithelial marker E‐cadherin (E‐cad) and gain of the mesenchymal marker Vimentin (Vim); epithelial cells are E‐cad^hi^/Vim^lo^ (red) whereas mesenchymal cells are E‐cad^lo^/Vim^hi^ (blue). E‐cad^hi^/Vim^hi^ (dark purple) and E‐cad^lo^/Vim^lo^ (light purple) cells are intermediate in this spectrum and constitute a hybrid epithelial/mesenchymal state, also known as “partial EMT.” (B, E) Percentage of total non‐targeting control (NT) (B) or *SOS2* ^*KO*^ (E) cultures treated with 150–300 nm osimertinib for up to 12 weeks that either did not become resistant (black) or developed osimertinib resistance. Osimertinib resistant populations are further stratified by E‐cadherin and Vimentin protein abundance as populations showing an epithelial (E‐cad^hi^/Vim^lo^, red), mesenchymal (E‐cad^lo^/Vim^hi^, blue), or hybrid E/M (E‐cad^hi^/Vim^hi^, dark purple or E‐cad^lo^/Vim^lo^, light purple) phenotype. (C, F) Quantification of E‐cadherin protein abundance, Vimentin protein abundance, and AKT phosphorylation from Western blotting experiments in osimertinib‐resistant NT (C) and *SOS2* ^*KO*^ (F) H1975 cell populations. Each dot represents an individual osimertinib‐resistant population; populations with high pAKT (closed circles) or low pAKT (open circles) are indicated. (D, G) Quantification of the percentage of E‐cad^hi^/Vim^lo^ (red), E‐cad^lo^/Vim^lo^ (light purple), E‐cad^hi^/Vim^hi^ (dark purple), or E‐cad^lo^/Vim^hi^ (blue) NT (D) or *SOS2* ^*KO*^ (G) populations showing high versus low pAKT levels. Comparison of the frequencies of pAKT^hi^ versus pAKT^low^ in D versus G was performed via contingency analysis in prism 9, and statistical significance was determined via chi‐square test; **χ ^2^ < 0.01.

EMT is a dynamic process by which epithelial cells acquire mesenchymal characteristics; the transition from epithelial to mesenchymal phenotypes can be characterized by the loss of E‐cadherin and an increase in Vimentin (Fig. 5A). Epithelial cells are E‐cad^hi^/Vim^lo^ whereas mesenchymal cells are E‐cad^lo^/Vim^hi^. Cells undergoing the epithelial‐to‐mesenchymal transformation can be either E‐cad^hi^/Vim^hi^ or E‐cad^lo^/Vim^lo^, although E‐Cad^hi^/Vim^hi^ is the most well characterized transitional state [17, 74, 75]. This hybrid epithelial/mesenchymal state, also known as partial EMT, is often seen in human cancers [74] and is associated with resistance to EGFR‐TKIs [13, 74, 75, 76, 77, 78].

We found that osimertinib‐resistant H1975 (NT) cells predominantly showed a hybrid E/M phenotype. Within the 66% of NT H1975 cultures that developed osimertinib resistance within 12 weeks, a majority showed a hybrid E/M phenotype that was predominantly E‐cad^hi^/Vim^hi^ (purple, Fig. 5B,C). The majority of osimertinib‐resistant cultures further showed elevated pAKT (Fig. 5D), with E‐Cad^hi^/Vim^hi^ hybrid E/M populations being enriched within the pAKT^hi^ cohort of resistant populations (Fig. 5C, closed circles and Fig. 5D). These data are consistent with previous studies showing that RTK‐dependent PI3K/AKT activation is a common hallmark of EGFR‐TKI resistance [52].

### 3.5 Osimertinib resistance via RTK/PI3K pathway reactivation is blocked by *SOS2* deletion

In contrast, within the 20% of *SOS2* ^*KO*^ cultures that developed osimertinib resistance within 12 weeks, the hybrid E/M pAKT^hi^ phenotype was much less prevalent (Fig. 5E,F). Instead, the majority of *SOS2* ^*KO*^ cultures able to develop osimertinib resistance did so by undergoing full EMT (E‐Cad^lo^/Vim^hi^, blue) and showed low pAKT (Fig. 5F, open circles and Fig. 5G). Notably, when accounting for the low frequency of osimertinib resistance that develops in *SOS2* ^*KO*^ cultures (66% NT versus 20% *SOS2* ^*KO*^), the percentage of osimertinib‐resistant E‐Cad^lo^/Vim^hi^ populations did not differ between NT (13%) and *SOS2* ^*KO*^ (12%) cultures (Fig. 5B,E). These data suggest that inhibiting proximal RTK signaling, achieved here via *SOS2* deletion, inhibits RTK/AKT‐dependent osimertinib resistance. These data further suggest that histologic transformation via EMT is an alternative pathway for osimertinib resistance distinct from RTK reactivation.

## 4 Discussion

Oncogenic *EGFR* driver mutations occur in 15–30% of lung adenocarcinomas [1, 2, 3, 4]. While treatment with the third‐generation EGFR‐TKI osimertinib enhances both progression‐free [5] and overall survival [6] compared to first‐generation EGFR‐TKIs and is the mainstay of therapy for these patients, resistance to osimertinib invariably emerges. Osimertinib resistance is most often driven by reactivation of RAS signaling via activation of multiple parallel RTKs [7, 8, 9, 10, 11, 12, 13, 14, 15, 16] so that single‐agent targeting of resistant tumors may be impractical [73]. To prolong the therapeutic window of osimertinib treatment we must identify secondary therapeutic targets whose inhibition either (a) enhances the initial efficacy of osimertinib, thereby reducing the overall tumor burden, or (b) inhibits the development of resistant tumor cells by targeting those pathways that drive resistance. Here, we show that the RASGEF SOS2 fulfills each of these criteria: SOS2 modulates the threshold of EGFR signaling to regulate proliferation of *EGFR*‐mutated tumors and *SOS2* deletion inhibits RTK/PI3K signaling to block osimertinib resistance driven by oncogenic shift to alternative RTKs.

Why does SOS2 deletion regulate mutant EGFR‐dependent AKT but not ERK signaling? We hypothesize that this is due, in part, to the markedly differing thresholds of EGFR stimulation required to fully activate the PI3K/AKT versus RAF/MEK/ERK cascades; small amounts of EGFR stimulation are needed to fully activate RAF/MEK/ERK signaling, whereas 5‐ to 10‐fold higher levels of EGFR stimulation are required to activate the PI3K/AKT pathway. Why might these differences be relevant to SOS1 and SOS2‐dependent signaling? Compared to other core components of the EGFR/RAS signaling pathway, the absolute abundances of SOS1 and SOS2 are extremely low [79], making SOS1/2 the “stoichiometric bottleneck” for signal transduction from EGFR to downstream effectors. Indeed, full ERK activation is observed with only 10% of the approximately 100 000 EGFR molecules engaged on a per‐cell basis, which corresponds to the 5000–10 000 SOS proteins (SOS1 + SOS2) available to transduce signaling from EGFR to RAS [79]. Coupling together the concepts of different thresholds of EGFR signaling being needed to activate ERK versus AKT with SOS protein abundance being the bottleneck for EGFR signal transduction, we hypothesize that changes in SOS protein abundance (SOS1 or SOS2) are likely to alter PI3K/AKT signaling to a greater extent than RAF/MEK/ERK signaling. Indeed, we and others have observed that *SOS2* ^*KO*^ inhibits RTK‐dependent AKT (but not ERK) phosphorylation in *EGFR*‐mutated (Figs 2, 3 and 5) and *KRAS*‐mutated cancer cell lines [25, 26] as well as in epidermal stem cells [32]. The selective SOS2‐dependent inhibition of AKT signaling may further explain the biphasic response to EGFR‐TKIs we observed in *Sos2* ^*−/−*^ MEFs (Fig. 1). AKT signaling is more important for survival of cells during oncogenic/transforming growth compared to proliferation of adherent cultures [25, 26]; the first osimertinib‐dependent decrease in cell number observed in *Sos2* ^*−/−*^ MEFs was due to a decrease in anchorage‐independent proliferation. Alternatively, the specificity of signaling from SOS2 to PI3K through RAS may also be due to colocalization of signaling components at the membrane, which has been proposed as a mechanism of regulation for RAS signaling [80, 81, 82, 83, 84]. Whether either of these mechanisms can fully explain the differential effect of SOS2 deletion on PI3K/AKT versus RAF/MEK/ERK pathway activation requires further study.

We previously showed that inhibition of proximal RTK signaling intermediates SOS1 or SHP2 synergistically enhanced the efficacy of osimertinib in short‐term (3–4 day) killing assays, but that *SOS2* deletion did not enhance osimertinib efficacy on this timescale [27]. These initial efficacy experiments, similar to most drug–drug synergy studies, were designed to assess secondary targets that would enhance drug‐dependent tumor killing but not necessarily inhibition of transforming growth. Further, most *EGFR‐*mutated LUAD cell lines grown in 3D require long‐term culture (2–3 weeks) to assess for differences in anchorage‐independent proliferation [27]. Here, we found that rather than altering transformation under the nutrient‐rich conditions used for most experiments, *SOS2* deletion reduced anchorage‐independent proliferation when EGFR/RTK stimulation was limiting in both MEFs (Fig. 1) and in *EGFR*‐mutated LUAD cell lines (Fig. 2). These data extend our original observations that in RTK/RAS mutated cancers [26, 27]; drug–drug synergy should be assessed under 3D culture conditions and suggest that one must also assess the effects of secondary therapeutic targets on multiple timescales to assess both inhibition of 3D spheroid survival (3–4 days) and proliferation (2–3 weeks).

In addition to enhancing the efficacy of an oncogene‐targeted therapy, an ideal co‐therapeutic would also delay the development of resistance, thereby enhancing the overall initial window of progression‐free survival for the patient receiving treatment. Reactivation of RAS signaling via mutation and/or amplification of multiple parallel RTKs is a common mechanism driving osimertinib resistance [7, 8, 9, 10, 11, 12, 13, 14, 15, 16], and RTK/RAS/PI3K signaling has been hypothesized as a convergent mechanism of EGFR‐TKI resistance [52]. SOS2 is critical for RTK‐RAS‐PI3K signaling in *KRAS*‐mutated LUAD cells [26] and *SOS2* ^*KO*^ reduced PI3K/AKT signaling in osimertinib‐treated cells (Fig. 2). Thus, we hypothesized that in addition to enhancing osimertinib efficacy, *SOS2* deletion would delay the onset of osimertinib resistance. To test this hypothesis, we used two distinct models of osimertinib resistance. Using a forced HGF/MET bypass model [62], *SOS2* deletion re‐sensitized HGF‐stimulated cells to osimertinib by inhibiting HGF‐stimulated PI3K signaling (Fig. 3), suggesting that reducing RTK‐RAS signaling is sufficient to limit resistance driven by oncogenic shift to an individual RTK. However, this type of “forced bypass” assay does not take into account the evolution cancer cells undergo during long‐term selection pressures whereby resistant tumors accrue multiple distinct resistance mechanisms [73].

To overcome these limitations, we developed an *in situ* resistance assay that models acquired resistance to RTK/RAS pathway inhibitors in large cohorts of cell populations [54]. Using this assay, we found that *SOS2* deletion reduced the overall frequency with which cultures developed osimertinib resistance (e.g., 66% NT versus 20% *SOS2* ^*KO*^ in H1975 cells, Figs 4 and 5). Osimertinib‐resistant populations isolated from *in situ* resistance assays showed resistance mechanisms similar to patient populations. The majority of resistant populations showed simultaneous hyperactivation of multiple RTKs [54] and reactivation of PI3K/AKT signaling (Fig. 5), whereas a minority of populations show histologic transformation via EMT (Fig. 5). In contrast, hybrid E/M cells with reactivated RTK/AKT signaling were almost absent from the pool of osimertinib resistant *SOS2* ^*KO*^ cultures. Instead, the few osimertinib‐resistant *SOS2* ^*KO*^ cultures that emerged did so primarily by undergoing non‐RTK/AKT‐dependent EMT (Fig. 5). Of note, the overall percentage of cultures able to become osimertinib‐resistant by undergoing full EMT (E‐Cad^lo^/Vim^hi^) did not differ between NT and *SOS2* ^*KO*^ conditions. These data suggest that targeting proximal RTK signaling has the potential to eliminate the majority of osimertinib resistance, since bypass RTK reactivation and/or tertiary *EGFR* mutations represent the majority of osimertinib‐resistant cancers [85].

In LUAD, RTK/RAS pathway reactivation and “oncogene addiction,” or the requirement to maintain elevated RTK/RAS/effector signaling, is not limited to *EGFR*‐mutated tumors [4, 53, 86, 87, 88]. Indeed, RTK pathway activation is a major resistance mechanism to oncogene‐targeted therapies in LUADs with EML‐ALK‐fusions [87, 89, 90], mutations in other RTKs (*NTRK1* [91], *ROS1* [92, 93], *RET* [94], *MET* [95], and *HER2* [96, 97]), or *KRAS* mutations [26, 98, 99, 100, 101, 102, 103, 104]. This addiction to RTK/RAS pathway signaling in LUAD suggests that inhibition of proximal RTK signaling is a potential strategy to limit resistance to targeted therapies in a majority of LUADs [4]. The SHP2 phosphatase acts as an adaptor to recruit SOS1 and SOS2 to RTK complexes [105, 106, 107, 108, 109]. Thus, in addition to SOS2, SHP2 and SOS1 are RTK signaling intermediates and potential therapeutic targets whose inhibition might limit resistance to RTK/RAS pathway inhibitors in LUAD. In addition to *SOS2* ^*KO*^, inhibition of proximal RTK signaling via the SHP2 inhibitors RMC‐4550 or SHP099 significantly inhibited osimertinib resistance in *EGFR*‐mutated LUAD cells [54]. The SOS1 inhibitor BI‐3406 significantly inhibited acquired resistance to KRAS^G12C^ inhibitors [110] or MEK inhibitors [29] in *KRAS* ^*G12*^‐mutated LUAD cells. Based on these data, we propose that inhibition of proximal RTK signaling could be a common mechanism to prevent resistance to targeted therapies in a majority of LUAD.

## 5 Conclusions

Our study expands on our previously outlined framework [27] for preclinical assessment of therapeutic combinations in *EGFR*‐mutated cancer cells. Not only do drug–drug synergy experiments need to be performed under 3D culture conditions, but combinations need to be assessed at multiple timeframes to determine the extent to which they enhance drug efficacy (3–4 days), limit oncogenic growth (2–3 weeks), and prevent acquired therapeutic resistance (6–12 weeks). Using this framework, we show that SOS2 fulfills the criteria of a secondary therapeutic target in *EGFR*‐mutated LUAD. *SOS2* ^*KO*^ enhanced the efficacy of osimertinib‐dependent inhibition of oncogenic (3D) growth and reduced the development of acquired osimertinib resistance by limiting RTK/PI3K pathway reactivation. These results, in conjunction with studies assessing SHP2 [54] and SOS1 [29, 110] inhibitors as secondary therapeutics in *EGFR*‐ and *KRAS*‐mutate LUAD, suggest that inhibiting proximal RTK signaling may be a common secondary therapeutic strategy to enhance outcomes for patients with RTK/RAS pathway mutated lung adenocarcinomas.

## Conflict of interest

The authors declare no conflict of interest.

## Author contributions

PLT and RLK designed the experiments and analyzed the data; PLT and RLK performed most of the experiments; AJL and KC performed Western blots and resistance assays; NES assisted with dose–response curves and Wester blots and gave conceptual input throughout the project; BRD assisted with dose–response curves and Western blots; JY assisted with analysis of resistant clones. PLT and RLK wrote the manuscript, NES and BRD edited the manuscript.

### Peer review

The peer review history for this article is available at https://www.webofscience.com/api/gateway/wos/peer‐review/10.1002/1878‐0261.13564.

## Supporting information


**Fig. S1.** The hybrid epithelial / mesenchymal (E/M) phenotype in osimertinib‐resistant cells is SOS2‐dependent.

## Data Availability

All data generated or analyzed during this study are included in the manuscript and supporting files. All primary data are available on request. All reagents are available from the Kortum laboratory and USUHS via an MTA.
