# Supplementary material for: SOS2 modulates the threshold of EGFR signaling to regulate osimertinib efficacy and resistance in lung adenocarcinoma
Source: Mol Oncol. 2024 Jan 18;18(3):641–61. doi: 10.1002/1878-0261.13564 (PMC10920089; doi:10.1002/1878-0261.13564)
Supplement: Supplementary file 1 — Fig. S1. The hybrid epithelial / mesenchymal (E/M) phenotype in osimertinib‐resistant cells is SOS2‐dependent. [file MOL2-18-641-s001.pdf]

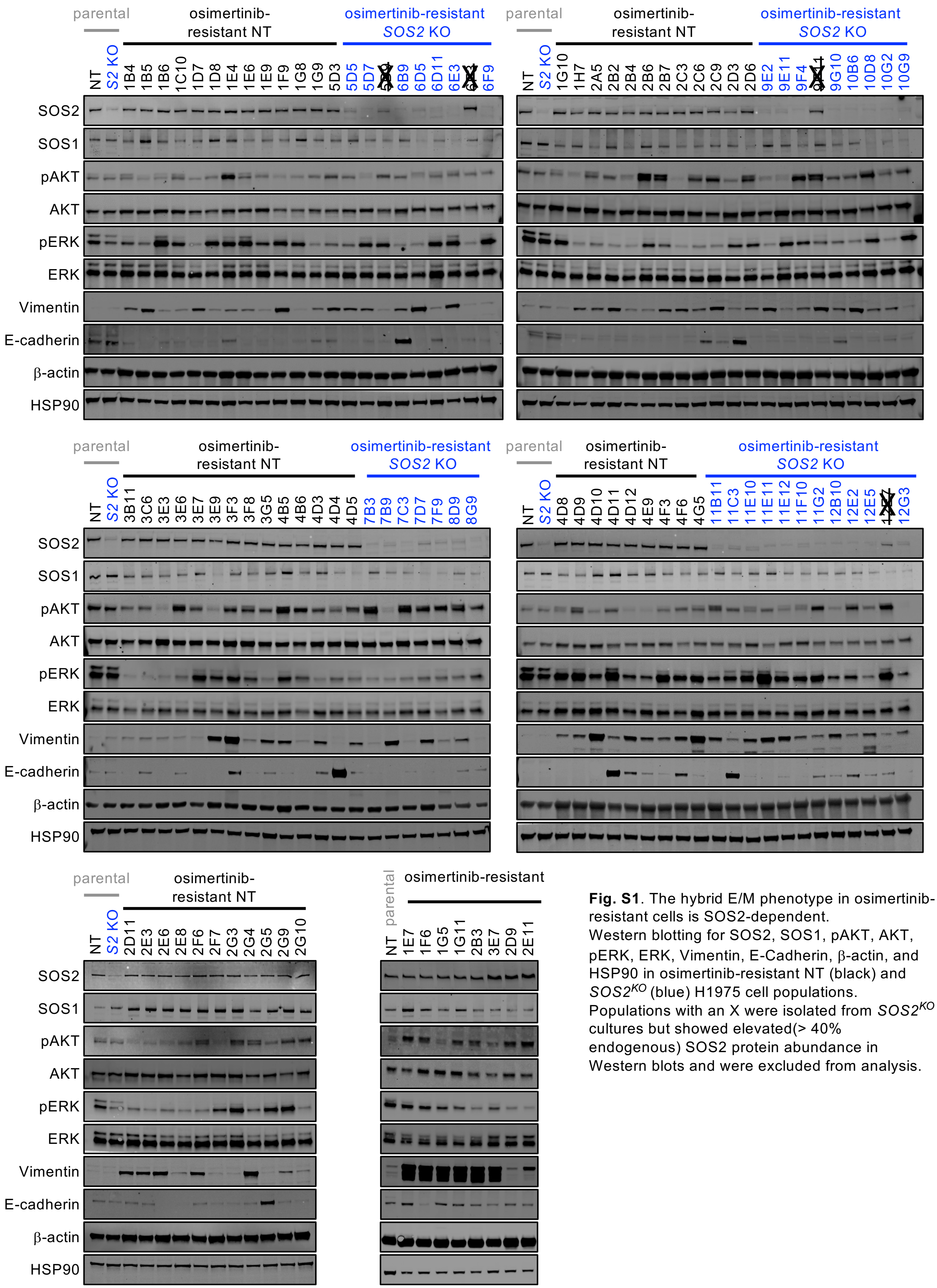

Fig. S1. The hybrid E/M phenotype in osimertinib-resistant cells is SOS2-dependent.
Western blotting for SOS2, SOS1, pAKT, AKT, pERK, ERK, Vimentin, E-Cadherin, β-actin, and HSP90 in osimertinib-resistant NT (black) and *SOS2*^{KO} (blue) H1975 cell populations. Populations with an X were isolated from *SOS2*^{KO} cultures but showed elevated(> 40% endogenous) SOS2 protein abundance in Western blots and were excluded from analysis.
